# A database of breast oncogenic specific siRNAs

**DOI:** 10.1038/s41598-017-08948-1

**Published:** 2017-08-18

**Authors:** Atul Tyagi, Manoj Semwal, Ashok Sharma

**Affiliations:** 1 Biotechnology Division, CSIR-Central Institute of Medicinal and Aromatic Plants, P.O.–CIMAP, Near Kukrail Picnic Spot, Lucknow, 226 015 Uttar Pradesh India; 2 ICT Division, CSIR-Central Institute of Medicinal and Aromatic Plants, P.O.–CIMAP, Near Kukrail Picnic Spot, Lucknow, 226 015 Uttar Pradesh India

## Abstract

Breast cancer is a serious problem causing the death of women across the world. At present, one of the major challenges is to design drugs to target breast cancer specific gene(s). RNA interference (RNAi) is an important technique for targeted gene silencing that may lead to promising novel therapeutic strategies for breast cancer. Therefore, identification of such molecules having high oncogene specificity is the need of the hour. Here, we have developed a database named as Breast Oncogenic Specific siRNAs (BOSS, http://bioinformatics.cimap.res.in/sharma/boss/) on the basis of the current research status on siRNA-mediated repression of oncogenes in different breast cancer cell lines. BOSS is a resource of experimentally validated breast oncogenic siRNAs, collected from research articles and patents published yet. The present database contains information on 865 breast oncogenic siRNA entries. Each entry provides comprehensive information of an siRNA that includes its name, sequence, target gene, type of cells, and inhibition value, etc. Additionally, some useful tools like siRNAMAP and BOSS BLAST were also developed and linked with the database. siRNAMAP can be used for the selection of best siRNA against a target gene while BOSS BLAST tool helps to locate the siRNA sequences in deferent oncogenes.

## Introduction

Breast cancer is one of the major cause of women death in India as well as throughout the world^1^. Breast cancer is a result of mutation in the genes involved in regulation of cell growth and proliferation^2–9^. In the process of breast cancer, genes are mutated which may result in gain- or loss-of-function that contribute to the malignant phenotype^8^. These mutations may be the consequence of spontaneous mutations, environmental factors, viral infection, etc. Anti-cancer drugs target the proteins encoded by these mutated oncogenes^3–11^. Amplification and overexpression of breast oncogenes are the major mechanisms through which these genes participate in the oncogenesis^4^. RNA interference (RNAi) was first reported by Fire *et al*. in *C. elegans* and has been used as a noble technique for cancer gene therapy^5^. Several studies have revealed the importance of short interfering RNAs (siRNAs) and short hairpin RNAs (shRNAs) in RNAi-mediated silencing of oncogenes as a potential therapeutic strategy for cancer^6, 7^. siRNAs are generally 19–25 nucleotides in length and have sequence specific gene knockdown capability. Reports on the basis of transfection of synthetic 21- and 22-nucleotide siRNAs with overhanging 3′ ends indicate that siRNA may be a powerful tool to suppress the target-specific gene expression^7^. In the last decade, several studies reported the use of numerous siRNAs and shRNAs for cancer gene therapy. There are few databases like DSTHO^8^, siRecords^9^, siRNAdb^10^, HuSiDa^11^, which focus on siRNAs targeting genes of human and other mammals. However, to the best of our knowledge, there is no comprehensive database of siRNAs/shRNAs targeting breast oncogenes which is required to search and analyse the data from the literature. With this in mind, a manually curated specialised databank, “BOSS”, has been developed on the basis of information from experimentally validated and published siRNAs and shRNAs targeting various breast oncogenes to facilitate research on RNAi-based cancer therapy. BOSS database maintains the comprehensive information of breast oncogenic specific siRNAs and comprises information about siRNAs name, target genes, inhibition value, cell line, siRNA sequence, NCBI Accession No., transfection reagent, test method, test objective and Pubmed ID etc. which are directly linked to essential databases. This database is an organised database where breast oncogenic specific siRNAs information is collected from literature and other existing databases to make it an informative tool for the researchers working in this field.

## Construction and Content

To develop a comprehensive database for breast oncogenic specific siRNAs, an extensive search was carried out to collect information on siRNA, shRNA and breast cancer. For this, first research articles and patents providing information related to breast oncogenic specific siRNAs were collected from various search engines like PubMed^12^ and Patent lens^13^. Specific searches were carried out using a combination of keywords like ‘siRNA’, ‘shRNA’, ‘Breast cancer’, ‘mammary cancer’, ‘cancer’, ‘gene therapy’, ‘gene silencing’ and ‘RNAi’. This exhaustive search yielded around 5613 research articles and 10 patents. From these articles and patents, only experimentally verified breast oncogenic specific siRNAs were retrieved manually. After careful reading of these articles, we scrutinised 88 research articles and 2 patents. Articles were carefully screened and about 865 siRNAs/shRNAs entries targeting breast oncogene with experimental studies were selected to be included in the database. Although most of the siRNA or shRNA entries have been provided with the inhibition value, qualitative representations were considered and included in the database if the quantitative values for siRNAs or shRNA were not given in the corresponding reports. These reports have evaluated the expression level of different test targets (i.e. mRNA, protein, etc) in breast cancer samples^14–25^. The most common method used to evaluate the efficacy was MTT assay. Different experimental methods like WST-8 Assay, RT-PCR, Western blotting etc. were used to validate breast oncogenic specifics siRNAs. Furthermore, 195 siRNAs derived from different assays have also been incorporated with information regarding their alternative efficacies.

## Database Architecture

**Figure 1:**
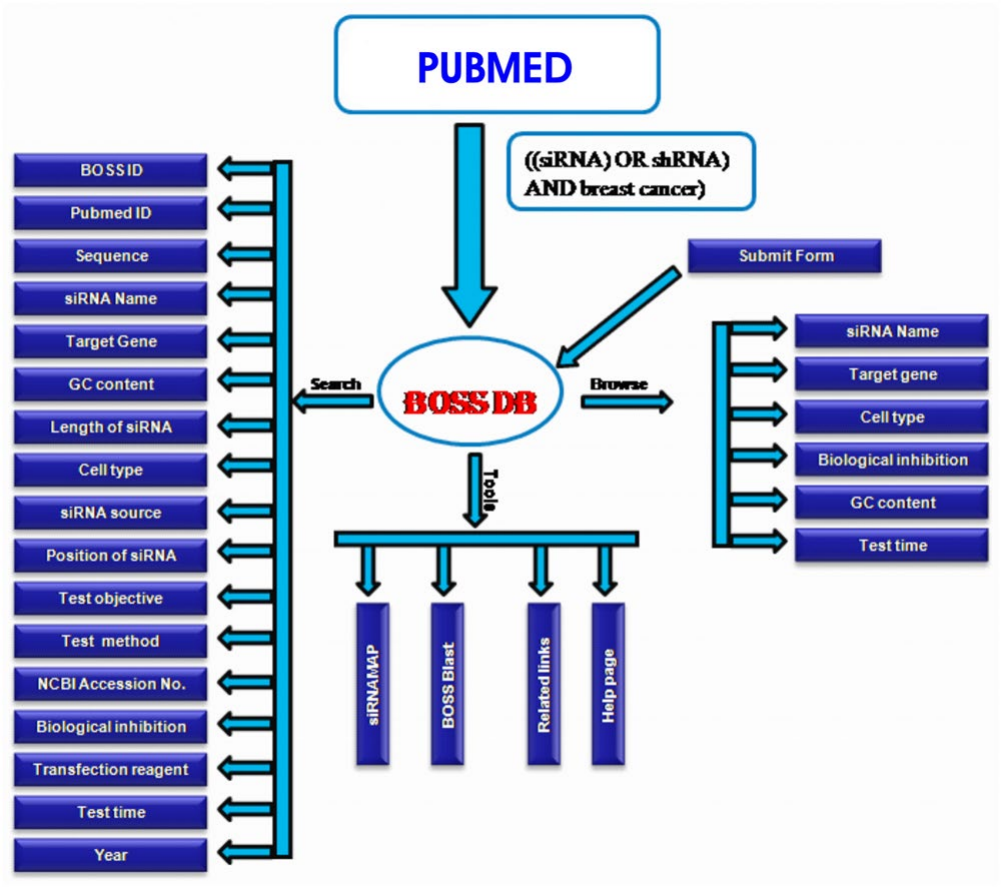
Systematic representation of BOSS database architecture.

As shown in Fig. 1, the BOSS database contains the following seventeen fields for each siRNAs entry; (1) BOSS id (2) PubMed id, (3) Sequence, (4) siRNA name, (5) Target gene, (6) GC content, (7) Length of siRNA, (8) Cell types, (9) Year, (10) siRNA source (siRNA/shRNA), (11) Position of siRNA, (12) Test objective, (13) Test method, (14) Gene Bank Accession No., (15) Biological inhibition, (16) Transfection Reagent and (17) Test time.

## Database Construction and Maintenance

### Database web interface and architecture

BOSS DB is built on Apache HTTP 2.4.9 and MySQL 5.6.17 Servers at the backend, whereas the front-end is built using HTML, PHP, jQuery, and Perl. MySQL is a management system for Open Source Relational SQL database. BOSS web interface and database interfacing scripts have been written in PHP, HTML, PERL, CSS and Java integration programming languages. BOSS database comprises diverse types of information about each siRNA entries, which is collected from different resources. Figure 1 shows the schematic representation of architecture of BOSS database.

## Utility

In order to facilitate the users’ search, several web-based tools have been integrated into the database that includes search, advance search and browsing options (Fig. 1).

### Search and advance search tool

The search tool is provided for searching all the seventeen fields or selected fields of the database. The advance search provides a refined way to search the database using several combinations of keywords of related fields using logical operators “AND” & “OR” for more specific results.

### Browse

A powerful browsing facility has been provided with BOSS database that allows users to browse data using various options. A short description of web interface designed for browsing are as follows:

This option allows the user to browse BOSS database based upon main categories like siRNA Name, Target Gene, Cell Type, Inhibition, GC Content and Test Time of breast oncogenic specific siRNAs. The user can select any of the categories and click the button to find a list of breast oncogenic specific siRNA related to the particular category.

## Web-Based Tools

### siRNAMAP

The siRNAMAP maps the BOSS database siRNAs on the basis of query nucleotide sequence. It will provide the list of siRNA from the BOSS database which is complementary to the of the user query sequences.

### Blast

This tool can be used for similarity-based search of any query sequences with those present in the BOSS database. By using this, user can examine whether a given breast oncogene-specific siRNA sequence or similar siRNA sequence has already been reported or not. The user can submit a query sequence in the Fasta format in the search field and press the submit button. It will display all breast oncogene-specific siRNAs similar to the query sequence. The server provides the option to modify different parameters like scoring matrices, gap penalty, word size etc^26^.

## Data Statistics and Findings

siRNA designing for knock-down target gene expression has been a major challenge. It is obvious from the earlier reports that only a fraction of designed siRNAs is highly effective in gene silencing^15–25, 27, 28^. Therefore, gene silencing experiments require a cost and labour-intensive optimisation protocol for designing and selection of efficient siRNAs and their delivery into the target cell lines. BOSS database consists of 865 entries for 195 unique breast oncogene-specific siRNAs/shRNAs. It comprises of diverse types of information about each siRNA entry, which is collected from the different resources. The database is an endeavour in the direction of RNA interference (RNAi) for breast cancer gene therapy. While searching for breast oncogene-specific siRNAs in the literature, it was observed that most siRNAs had been tested on various breast cancer cell lines showing different biological inhibition values. The percent efficacy ranges from 0–100 in BOSS database. Negative efficacy values reported in a particular experiment with respect to control was considered as zero, for the sake of simplicity.

**Figure 2:**
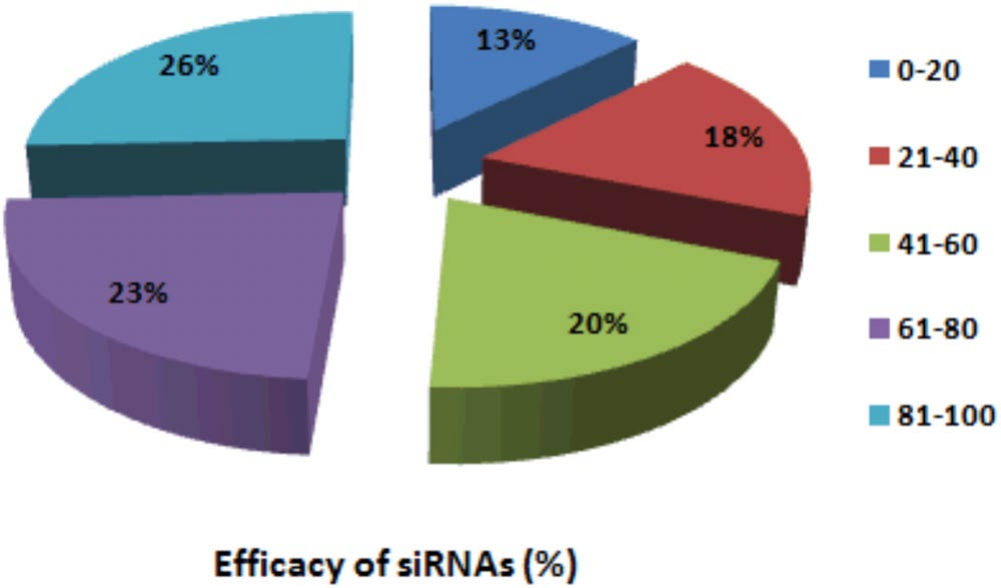
Percentage of different si/shRNAs included in the database suppressing the target gene expression to different levels (0–100%).

As shown in Fig. 2, number of si/shRNAs with inhibition percentage 81–100, 61–80, 41–60, 21–40 and 0–20, were 26%, 23%, 20%, 18% and 13%, respectively.

**Figure 3:**
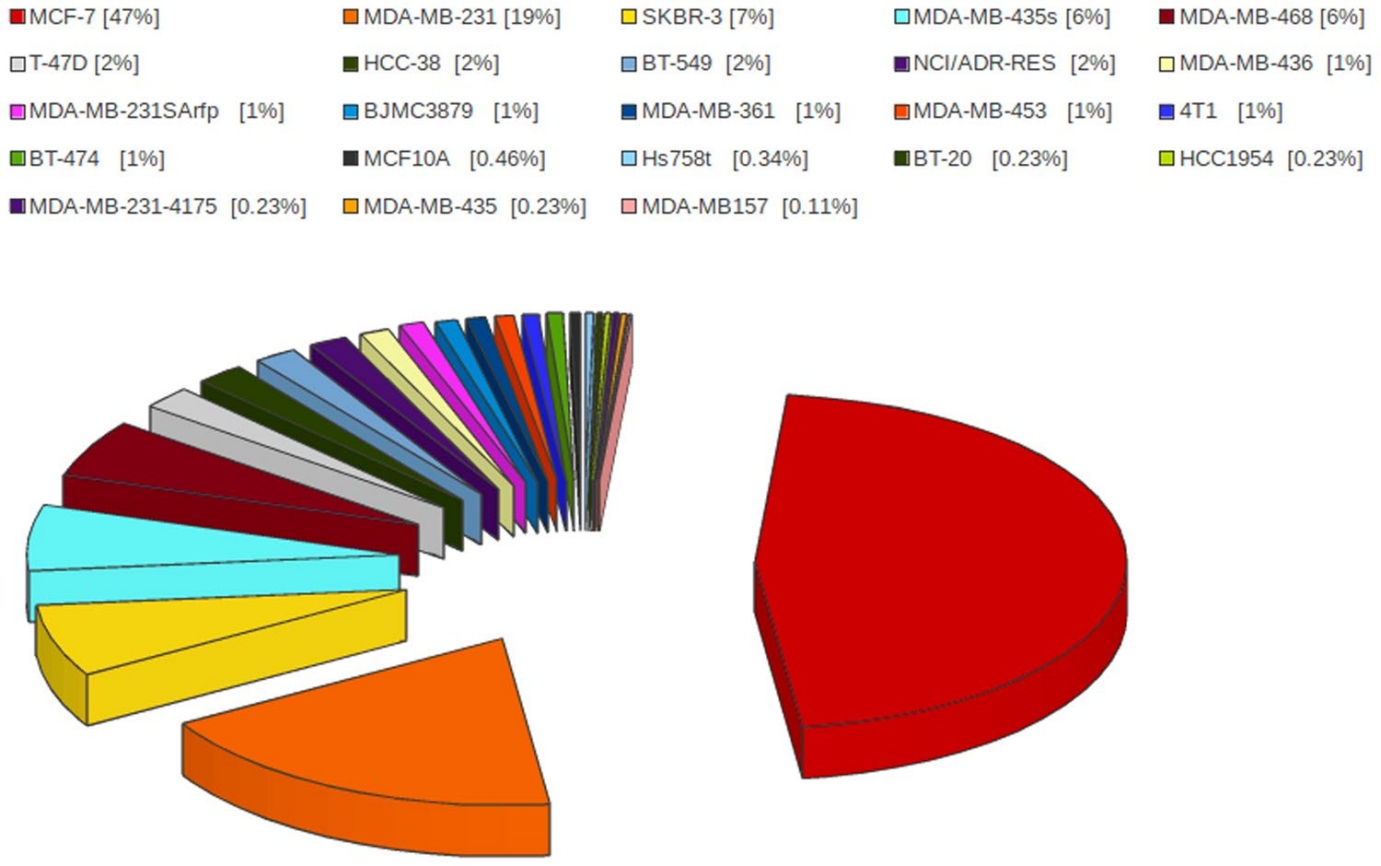
Cell lines used to examine siRNA-mediated suppression of different oncogenes.

The database entries contain siRNA experimentally validated using 23 different cell lines but MCF-7, MDA-MB-231, SKBR-3, MDA-MB-435s and MDA-MB-468 cell lines were mostly used. Different cell lines used to examine siRNA-mediated suppression of different oncogenes are given in supplementary information. There are a number of siRNAs in our database, whose breast oncogenic activity (knockdown efficacy) has already been tested against several types of breast cancer cell lines. Although MCF-7 and MDA-MB-231 were used in most of the cases, this database has siRNA entries targeting a breast oncogene in all the reported breast cancer cell lines. The overall statistics of the different cell lines is depicted in Fig. 3. MCF-7 and MDA-MD-231 lines were used for 47% and 19% siRNAs entries, respectively.

Percentage of different si/shRNAs included in the database suppressing the target gene expression.

**Figure 4:**
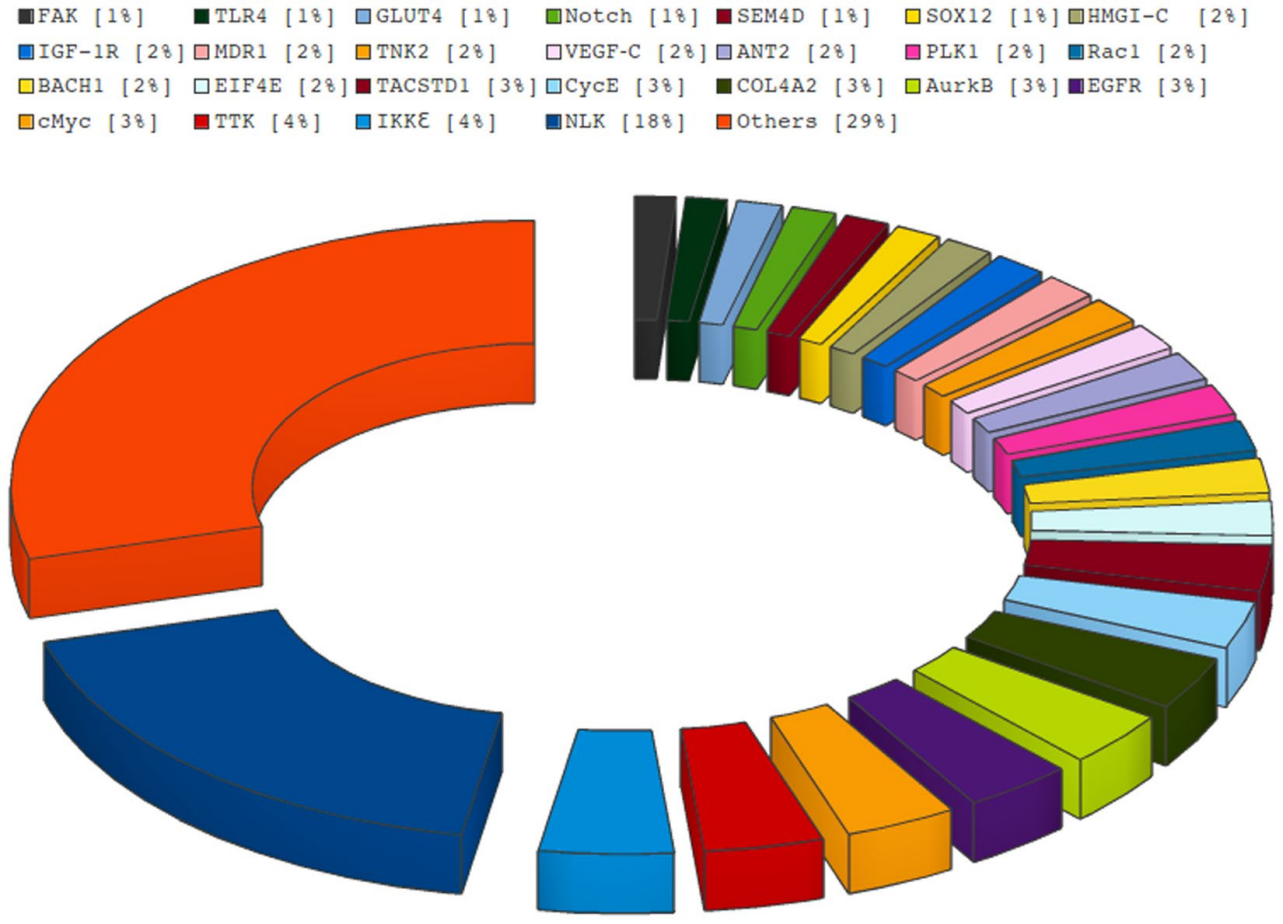
Oncogenes targeted by siRNA-mediated suppression.

The BOSS database provides sequences of reported functional siRNAs targeting breast oncogenes and other technical details of the corresponding experiments, including used cell lines, transfection reagents and direct links from the published references. Out of 865 entries, 477 entries were found in Genbank database that lies in 37 different breast genome regions. Users can explore information about the siRNAs/shRNAs sequences, target Homo sapiens genome region, efficacies and the experimental conditions prior to their experiments in user-friendly manner using the search and browsing facility. BOSS database provides experimentally validated siRNAs reported in literature targeting diverse genes of Homo sapiens genome region. The majority of the reported breast oncogenes targeted by RNAi-mediated suppression were NLK (18%), IKKƐ (4%), TTK32 (4%), EGFR (3%) and AurkB (3%) respectively (Fig. 4). Different breast oncogenes targeted by siRNA-mediated suppression are given in supplementary information. The transfection reagents oligofectamine and lipofectamine covers 18% and 35% of the database, respectively. G + C content is the crucial character for functional siRNAs^29^. The G + C content profile was used for visualisation and analysing the variation of GC content in genomic sequences^30^. DNA molecules were made up of four nitrogenous bases A, T, G, C. These four nitrogenous bases made different number of hydrogen bonds with each other. Due to three H-bonds between G and C, this base pair is stronger than that of A and T. This makes high G + C containing DNA thermally more stable than AT containing DNA^29^. It has been reported that sequences of intermediate G + C contents (around 50%) were more effective siRNAs, and our dataset contained 86% of siRNA in the intermediate range (30–65%) G + C content^31^.

## Discussion

### Comparison with other databases

Very few databases of siRNAs are available viz., DSTHO^8^, siRecords^9^, siRNAdb^10^, HuSiDa^11^, which focus on siRNAs targeting genes of both human and other mammals. HuSiDa and siRNAdb are based on published functional siRNA targeting human genes. DSTHO is based on human oncogenes but not for breast oncogenes. The lack of updates and comprehensiveness is another problem with this database^8^. The present database on breast oncogenic specific siRNAs will be useful in designing and/or evaluating the breast oncogene-specific si/shRNAs, such as VIRsiRNAdb^32^ database which is specific for experimentally validated viral-specific siRNA/shRNA. In our earlier study, we have also developed, curated and developed a database named HIVsirDB, HIV specific siRNAs against HIV^28^.

## Conclusion

siRNA has been proven to be a valuable tool for knocking down the expression of specific human genes. siRNA exhibit a high degree of specificity and has important medical implementations such as oncogene repression in cancer_._ The uniqueness of the system, that makes it a powerful tool is the sequence specificity towards a particular gene. It is fast, easy and the most cost-effective processing. This oncogene-specific RNA interference will offer plenty of opportunities for the researchers in exploring the role of breast oncogene specific siRNAs in breast cancer. In addition, siRNA designing algorithms also help to make effective molecules. To the best of the authors’ knowledge, no such database is available for breast oncogene siRNA.

## Electronic supplementary material

Supplementary Dataset 1

Supplementary Dataset 2
